# Summary of approach to inclusive language in vaccine guidance from the National Advisory Committee on Immunization (NACI)

**DOI:** 10.14745/ccdr.v52i04a01

**Published:** 2026-04-30

**Authors:** Ana Howarth, Matthew Tunis, Christina Jensen, Stephie Pierre, Fiann Crane, Winnie Siu, Robyn Harrison

**Affiliations:** 1 Centre for Immunization Surveillance and Programs, Public Health Agency of Canada, Ottawa, ON

**Keywords:** National Advisory Committee on Immunization, inclusive language, pregnancy, breastfeeding, gender, representation, equity

## Abstract

**Background:**

The National Advisory Committee on Immunization (NACI) has been working to develop inclusive language for vaccine guidance related to pregnancy and breastfeeding. Starting with early efforts in 2018, NACI sought to update the language approach in 2023 based on the latest available evidence.

**Methods:**

Gender-neutral and gender-additive language approaches were assessed using an equity lens focused on inclusion and representation. Evidence was gathered through a jurisdictional scan of National Immunization Technical Advisory Groups (NITAGs) and government organizations, a literature review, and iterative stakeholder engagement. Draft policy options were developed, translated into French, and reviewed by NACI.

**Results:**

The jurisdictional scan across Canada, the United States, Australia, the United Kingdom, and France showed wide variation, with many sources using gendered terms. The literature review identified little original research on language use in immunization settings. A Canadian research initiative examining inclusive language supported context-specific gender-additive terminology. Stakeholders emphasized the need for inclusive and adaptable language. NACI developed recommendations for a context-dependent gender-additive approach, including French language options.

**Conclusion:**

In February 2024, NACI endorsed an additive language approach, which has been implemented across NACI statements and relevant Canadian Immunization Guide chapters. NACI and the Public Health Agency of Canada will continue adapting to evolving language needs, recognizing that clinical communication should use terminology that best resonates with each individual. When implementing vaccine recommendations in clinical practice, it is important to use whatever language that will resonate best with the individual being immunized.

## Introduction

Appropriate descriptions for pregnant women and pregnant individuals have been under consideration for many years by the National Advisory Committee on Immunization (NACI) as the guidelines produced aim to be inclusive of pregnant individuals who may not identify as women, including transgender and non-binary individuals. These discussions initially started in 2018 with NACI’s advice on the tetanus, diphtheria and acellular pertussis vaccine in pregnancy ((1)). At the time, NACI issued a note in the statement to acknowledge the need for inclusion, “NACI recognizes that not all people giving birth will identify as women or mothers. For the purposes of this statement, the terms “pregnant woman”, “mothers” and “maternal” are used, but should be considered to also apply to those individuals who do not specifically identify as female gender but are the parent gestating the fetus.” ((1)).

With several ongoing NACI working groups actively reviewing immunization guidance in pregnancy (COVID-19, influenza, respiratory syncytial virus) and due to the lack of a standard approach to language in pregnancy, NACI working groups were having parallel and similar discussions about appropriate representative terms to foster inclusivity.

To address this issue, in 2023 the NACI Secretariat created a technical group tasked with identifying standardized inclusive language that could meet expectations of diverse stakeholders and be pragmatic to use (i.e., not distracting from the communication goals and overall usability of vaccine guidelines). Research has shown that language is a powerful tool that can be used to improve health outcomes when it replaces existing discriminatory linguistic practices ((2)). This initiative was launched to develop a language approach to pregnancy and breastfeeding for NACI guidance and communication that best reflects the key concepts of equity, inclusion and representation, which support the NACI mandate ((3)). More specifically, the aim of this project was to review evidence, consult key stakeholders, including subject matter experts (i.e., as outlined in the sections below), and to establish guidance on language for interim use until a more global consensus is reached. The key deliverables that were created to meet this overall aim were 1) a policy statement on the use of language in clinical guidance about immunization during pregnancy and breastfeeding, 2) notices for publications and 3) a bilingual lexicon with standard terms.

## Methods

Gender-neutral language and gender-additive language were assessed as options. Gender-neutral language avoids referencing a specific sex and gender (e.g., pregnant people or pregnant individuals), gender-additive language uses the term “women” alongside gender-neutral language (e.g., pregnant women and pregnant people or pregnant women and pregnant individuals) to achieve both inclusivity (i.e., for those who are pregnant but who do not identify as women) and representation (i.e., for cisgendered women who as an existing equity group still need representation). Use of anatomy-based language was not assessed because of evidence identification of people by their body parts can be dehumanizing ((4)). Challenges with gender-neutral language have been identified by various equity stakeholders working within research and health care ((5–7)).

Evidence-gathering activities included a jurisdictional scan of National Immunization Technical Advisory Groups (NITAGs), government organizations (i.e., international, national and provincial) and relevant organizations/societies across jurisdictions, a targeted literature review, and both internal and external stakeholder engagement. Stakeholder engagement was an iterative process of incorporating feedback after each engagement to build on the evidence gathered and allow for subsequent engagement best reflecting real-world feedback.

Based on the combined results of evidence gathering activities, initial draft guidance and a draft policy were developed. French language translation was then undertaken by francophone members of the research group in collaboration with francophone colleagues from the Public Health Agency of Canada (PHAC) and Health Canada who had relevant expertise or lived experience. Following this, a proposed policy statement with recommendations for NACI to adopt and implement was prepared and presented to NACI for approval on February 23, 2024.

## Results

### Jurisdictional scan

A scan was conducted on approaches to gendered language used in the five key jurisdictions (i.e., Canada, United States [US], Australia, United Kingdom and France) with languages deemed most relevant to the general population of Canada. A further sub-scan of provinces and territories, including *ad hoc* women’s health organizations and obstetrics and gynecological societies, was also included. Results across jurisdictions were varied but gendered language (e.g., pregnant women) appeared most commonly and no jurisdictions were found to use gender-neutral language for breastfeeding (e.g., chestfeeding). Several health organizations such as Health Canada, PHAC and the US Centers for Disease Control and Prevention were found to have used gender-neutral language and one organization (i.e., England’s National Institute for Health and Care Excellence) used gender-additive language (see **Appendix** for overview). No national agencies or regulatory bodies were found to use gender-neutral language for breastfeeding but a scan of four key lactation associations (within Canada and the US) found that three of the four utilized gender-neutral language.

Three out of the seven agencies reviewed were found to have some version of policy statement or guidance regarding the use of gender-neutral language for the purpose of inclusivity; however, across five key Canadian and US obstetrics and gynecological/midwifery organizations there were many instances of the use of gender-neutral language for pregnancy and one for breastfeeding and all had some form of statement or guidance for inclusive language (see examples below).

Examples from language statements:

“The SOGC [Society of Obstetricians and Gynaecologists of Canada] recognizes the importance to be fully inclusive and when context is appropriate, gender-neutral language will be used. In other circumstances, we continue to use gendered language because of our mission to advance women’s health.” – Society of Obstetricians and Gynaecologists of Canada

“To be inclusive of women and all patients in need of obstetric and gynecologic care, ACOG [American College of Obstetricians and Gynecologists] will move beyond the exclusive use of gendered language and definitions.” – American College of Obstetricians and Gynecologists

“In our language we will aim to add and not take away, taking into account the importance of preserving women-centred language as well as including language for those who do not identify as a woman.” – Royal College of Obstetricians and Gynaecologists

In summary, the jurisdictional scan highlighted several key findings, including the identification of Beyond the Binary BC (BTB BC), which was a provincial research project focused specifically on developing gender equitable language practices within research framed as “women’s health”. This two-year project involved extensive stakeholder engagement, including collaboration with both a community and research committee, and eventual Canadian Institutes of Health Research funding to scale the work to a national level. Beyond the Binary BC‘s approach advocated for context-specific language and, where feasible, introduces a gender-additive approach. Most recently (October 2024), the BTB Canada Guide ((8)) was launched after a pan-Canadian Research Task Force and Community Task Force had been convened. The resulting guide aims to offer “a nationally relevant, and feasible guidance and resource package to support health researchers and health research institutions in their commitments to conducting gender equitable health research for women, trans, and non-binary people”. Beyond the Binary BC’s work and the BTB Canada Guide provides support and a rationale for an additive language approach, where feasible, as evidenced from those with lived and professional experience in this area.

### Literature review findings summary

A targeted literature review was conducted in July 2023, with the inclusion criteria of peer-reviewed articles published from English-speaking high-income countries and France. Articles published five or fewer years ago with a focus on gender and language within a pregnancy and/or lactation context were included. A total of 12 articles were included in the final selection, of which most (including a conference abstract) were commentaries or editorials.

Of the included studies, only one was an original research article, where acceptability (and understanding) of gender-neutral language in a Breastfeeding Attrition Prediction Tool was tested with a sample of 16 participants. Most articles (n=10) aligned with a certain viewpoint or stance in relation to language: half of the studies advocated for an exclusively gender-neutral approach; four articles supported the use of both sexed language and gender-neutral language; one article was specific to a trans/nonbinary population only; and one article did not confirm a language approach but emphasized the importance of considering language use and how it may impact those involved.

Of the seven articles (see **Table 1**) that offered proposed language options, all were gender-neutral recommendations and only one article ((9)) included mention of women and the issue of representation. Many articles were focused on addressing inclusivity with less than half (n=5) including a discussion of the issue of erasure of women, existing women’s inequity within health care or representation of women within the context of using gender-neutral language; however, a key limitation of the literature review was the lack of original research suggesting a gap in the knowledge base of evidence for feasible and acceptable language approaches for those specifically impacted.

**Table 1:** Studies with proposed language options and summary of the position they present (n=7)

Primary author, year of publication	Pregnancy and/or lactation language	Mention of women/ representation	Summary of position
Duckett *et al.*, 2019 (10)	Birthing parent Breastfeeding Chestfeeding	No	Some solutions are offered, mostly gender-neutral in nature. Option of having different sets of materials for cisgender parents and those who identify as LGBTQI+ considered.
Parker *et al.*, 2023 (9)	Pregnant or birthing people Breast/chestfeeding	Yes	The critical role of midwifery education/educators in taking up the challenge of inclusion to ensure a workforce skilled and supported in the provision of care to a gender diverse population is affirmed but solutions are not specifically offered.
Dinour, 2019 (11)	Gestational parent Birthing parent Feeding at the breast/chest At-the-breast/chestfeeding	No	Gender-neutral is recommended—several nongendered terms drawn from ILCA guidelines, published research and/or guidance from individuals or groups working with LGBTQIA+ communities.
Dodgson *et al.*, 2022 (12)	Birth parent or mother Self-identified mothers Persons self-identifying as women Persons identifying as the primary caregiver Breastfeeding and lactation	No	Gender inclusivity is recommended—to better align language with “movements towards greater health equity and social and publication ethics”.
Kinney *et al.*, 2023 (13)	Pregnant people Chestfeeding Bodyfeeding	No	Language should be revised to be more gender inclusive but also options should be tested with target populations as more research is needed.
Rioux *et al.*, 2022 (14)	Pregnant people Pregnant individuals Chestfeeding Human milk feeding Nursing	No	A shift to gender inclusive language should be seen as a priority for research on pregnant populations.
Roosevelt *et al.*, 2021 (15)	Pregnant people Chestfeeding	No	As focus is specific to the clinical treatment of trans/nonbinary people, recommendations are for individualized care and use of gendered-affirming language.

Abbreviations: ILCA, International Lactation Consultant Association; LGBTQI+, lesbian, gay, bisexual, trans, queer/questioning, intersex, and other; LGBTQIA+, lesbian, gay, bisexual, trans, queer/questioning, intersex, asexual and other

### Stakeholder engagement findings

Internal engagement within PHAC and Health Canada, external engagement, and consultation with an expert pregnancy immunization group were undertaken to collect real-world information in relation to impacted communities and individuals.

**Government of Canada’s Internal Stakeholder Engagement:** Internal engagement was undertaken with teams from PHAC and Health Canada including the Sex and Gender-Based Analysis Plus team, the Gender and Sexual Diversity Network and subject matter experts from other PHAC and Health Canada teams (e.g., Maternal and Child Health, Sexual and Reproductive Health) who offered both professional and lived experience input. Key takeaways from PHAC internal engagement included the following:

· There is a need for appropriate and inclusive language that would be implementable across a variety of communication contexts (e.g., public facing, scientific reporting, journal/chapter editing).

· Public Health Agency of Canada researchers working with Indigenous women’s organizations reported that they had received feedback from an Indigenous women’s group who thought the use of gender-neutral language was removing their lived experiences as women from the conversation, something they had worked hard to achieve. The researchers’ solution for the research communication being designed was to use “women and people assigned female at birth”.

· Some participants expressed concern that additive or sexed language may alienate or “other” gender diverse individuals who can be included in gender-neutral approaches.

· An overall theme was that it is critical to consider context when choosing an approach.

**External Stakeholder Engagement:** External engagement included representatives from the Campbell and Cochrane Equity Methods Group and co-chairs and leads involved with BTB BC ((4)) project, which was identified through the jurisdictional scan. Key takeaways from external engagement included the following:

· Co-chairs and leads involved with the BTB BC project have been working intensely for the past few years on producing an inclusive language guide and glossary. By systematically reviewing the literature and working with groups representing community members, clinicians and researchers, they concluded that an additive approach (depending on context) is a valid option for an inclusive strategy. Beyond the Binary Canada (published and available as of October 2024) is conducting ongoing national research with the intention of providing an inclusive language guide in both English and French.

· Using only a gender-neutral approach (e.g., individuals with a cervix, lactating, birthing, etc.) can inadvertently lead to the erasure and exclusion of women and especially subgroups of women, such as Indigenous, Black and other racialized women, who already experience inequity.

· Using a gender-neutral approach can be confusing for some newcomers to Canada when English is not their first language and if they do not see themselves in the gender-neutral language.

· Consistent with internal engagement feedback, context was reported as very important to consider when choosing an approach.

**Expert Pregnancy Group Consultation:** NACI Working Groups often consult Canadian pregnancy experts when developing guidance on vaccination during pregnancy. In this case, Canadian pregnancy experts were consulted to contextualize language findings. Based on the available evidence, there was strong support for the use of the additive language approach as presented (with the exception of breastfeeding), as opposed to the gender-neutral approach that could lead to excluding women and their experiences. Consultation identified that:

· Certain terms may be triggering to some populations (e.g., breasts, breastfeeding) and this should be mitigated where possible.

· The importance of scientific accuracy versus the desire to be inclusive through the use of gender-neutral language could lead to uncertainty; for example, the word “chestfeeding” instead of “breastfeeding” may not clearly identify the source of the milk when the transfer of antibodies is an important consideration for passive immunization.

· It is important to include sensitivity statements when using certain terms, particularly where the scientifically accurate term is the most appropriate option.

· Pregnancy experts suggested that it would be appropriate to add a notice in the preamble before certain NACI (or NACI Working Group) meetings/presentations, emphasizing that language using pregnant women and/or breastfeeding may be in reference to those who do not identify as women but are pregnant.

### French translation

All NACI guidelines are published in both official languages, so any English language approaches must also be consistent in French. An informal environmental scan of web content in French (e.g., government websites, community and women’s health organizations/obstetrics and gynecological societies) and selection of suitable terms for pregnancy and breastfeeding was conducted. Most websites used gendered language (e.g., “femme enceinte”). Some web resources used gender-neutral terms, such as “personne enceinte”. Additionally, in French, the term “allaitement” (meaning breastfeeding) is gender-neutral and refers specifically to the act of milk delivery, regardless of gender. Based on these findings and PHAC translation expertise, a translation was developed. Consultation and feedback were then solicited from two French-speaking subject matter experts from Health Canada and PHAC.

### Language approach

The language options aimed to ensure that everyone is represented and included in vaccine advice. In this instance, it means applying a gender-additive approach where the term “woman” is used alongside gender-neutral language. This is intended to demonstrate a commitment to redress the historic exclusion of trans and non-binary people, whilst avoiding the risk of marginalizing or erasing the experiences of women within the healthcare environment. Communication must consider the audience being engaged. Different approaches are beneficial across different contexts; therefore, two approaches have been developed: a default approach and a tailored approach.

The default approach is where additive language is universally adopted across all contexts related to pregnancy and breastfeeding to provide accurate and consistent language. In addition, a notice accompanies this approach to explain the definition and rationale of additive language, with the aim of promoting understanding and fostering inclusivity and acceptance. See **Box 1** below for the final NACI policy statement on inclusive language for pregnancy and breastfeeding in immunization.

**Table:** Box 1: National Advisory Committee on Immunization policy statement on inclusive language approach to pregnancy and breastfeeding in immunization

**NACI approach to language in pregnancy and breastfeeding: Gender-additive language policy statement** **Appropriate descriptions for individuals who are pregnant or breastfeeding have been under consideration for many years within the NACI Secretariat and NACI working groups where the vaccine guidelines need to be inclusive of individuals who may not identify as women, including transgender, non-binary and gender fluid individuals.** **Research has shown that language is a powerful tool that can be used to improve health outcomes if designed to address existing discriminatory linguistic practices.** **Following a comprehensive approach to gather information including a jurisdictional scan, literature review, stakeholder engagement and expert consultation, NACI concluded that the adoption of gender-additive language best reflects the key concepts of equity, inclusion and representation at this time. Gender-additive language is language that uses the term “women” along with gender-neutral language (e.g., pregnant women and pregnant individuals). Unlike gender-neutral language, gender-additive language does not inadvertently perpetuate the erasure and exclusion of women. Certain groups of women (e.g., Indigenous, Black and other racialized women) already experience health inequities and gender-neutral language may exacerbate such inequities by impacting communication around issues affecting them. Gender-neutral language may also be confusing for newcomers to Canada, who may not see themselves represented in the gender-neutral language.** **Moving forward, NACI’s guidance, CIG and other resources and communications from the NACI Secretariat will use gender-additive language where possible to recognize and affirm all people who seek information on immunization, whether that be for themselves, their clients or their communities.** **NACI recognizes the limitations of applying gender-additive language when source material, such as published primary research studies, use gender-binary terms to describe data. NACI’s guidance will continue to describe data based on the terms reported by study investigators and researchers. In these contexts, when using gender-additive language is not accurate, a notice will preface the statement, literature review or CIG chapter, to address the topic of language inclusivity and provide an explanation for the lack of gender-additive language (i.e., that gender-additive language would not be reflective of the language of the study). Additionally, a message at the beginning of publications may be added to introduce the concept of additive language where appropriate. Some healthcare providers may not be familiar with additive language and the importance of inclusion of trans and non-binary populations.** **NACI and PHAC acknowledge the dynamic nature of language in society, and the fact that appropriate representations will likely change and evolve over coming years.**

Abbreviations: CIG, Canadian Immunization Guide; NACI, National Advisory Committee on Immunization; PHAC, Public Health Agency of Canada

The tailored approach is ideal for situations where additive language may not be feasible, and a more nuanced strategy is required. With this option, language is tailored to ensure comprehension and accessibility. Sensitivity to diverse experiences remains a priority, even if not explicitly reflected by the language used. These language approaches are detailed in the Appendix, according to context. It is acknowledged that, for knowledge translation and communication materials for the general public, use of second person language may be most appropriate to ensure clarity. As an example: “If you are pregnant or are planning to become pregnant, this guide is for you! Having a baby can be a wonderful experience…”.

## Conclusion

In February 2024, an additive language approach was proposed to NACI and was approved by the committee. Following this, the draft guidance and policy statement were finalized and have been implemented across recent NACI statements, recently updated Canadian Immunization Guide chapters and other NACI-related content.

It is hoped that the development of this pragmatic approach for interim use, in the absence of global consensus, will continue to advance key concepts of equity, inclusion and representation that support the NACI mandate. This dedicated evidence review and stakeholder consultation, together with the resultant policy statement, also facilitated current NACI goals for efficient, evidence based and timely publications with clear communication and harmonized publication products (such as the Canadian Immunization Guide). The products serve diverse stakeholder groups, all with the shared aim of improving health outcomes.

One challenge is the inevitable reliance of NACI content on scientific evidence, where inclusive language cannot be consistently applied. Current research practice, which has a history of focusing on cisgender men, has tended towards a binary approach with sex being reported but not gender. Innovators in this area will hopefully move forward with options for health researchers that adapt to context and equity considerations such as intersectionality, allowing for all individuals, communities and stakeholders to see themselves reflected in language and research findings.

NACI and PHAC will continue to strive to bridge research, public health, clinical practice and collective experiences in ways that best serve and care for all individuals and communities in Canada. It is acknowledged that language, like culture, evolves, and understanding context (i.e., audience, setting and purpose) is key. With the next iteration in refining this language, there may be opportunities to explore novel methods to engage effectively with key populations such as individuals from First Nations, Inuit, and Métis communities and individuals from 2SLGBTQI+ (two-spirit, lesbian, gay, bisexual, trans, queer. intersex, and additional people who identify as part of sexual and gender diverse communities) and other communities. When implementing vaccine recommendations in clinical practice, it is important to use whatever language that will resonate best with the individual seeking immunization.

## Appendix

**Table A1:** Language approaches according to context

**Default Approach:** Use of gender-additive language with a notice that introduces additive language and rationale; notes the dynamic nature of language and exceptions for instances where primary research is reported.			
**WHEN TO USE…** NACI Statements, *Canada Communicable Disease Report* (CCDR) articles or CIG chapters			
Try this…	Instead of this…		Because…
Pregnant women and pregnant people Pregnant women and pregnant individuals	Pregnant women Pregnant people Pregnant persons Pregnant individuals		A move to the additive use of gender-additive language (i.e., where gender-neutral language is used alongside the language of womanhood) ensures that everyone is represented and included. The use of “pregnant” prior to each group/person mentioned is necessary to avoid confusion.
	As an example: “This statement addresses **Tdap immunization of pregnant women and pregnant people** in Canada with the aim of protecting newborn infants in Canada from severe outcomes of pertussis infection”.		
**Breastfeeding and maternal** It is acknowledged that sometimes there is no general consensus or appropriate language option currently available, and that this terminology requires more development. In English, for breastfeeding, the option of “chestfeeding” is one possible proposed term for those (e.g., trans people or nonbinary people) who may feel words like breastfeeding or nursing are not the right fit because the language does not align with their gender or how they identify their anatomy. However, when discussing breastfeeding or breastmilk specifically in terms of antibody transfer or the transfer of a pathogen via breastmilk, it may not be appropriate to use the term “chestfeeding” as the biological basis may be unclear. As the word for breastfeeding in French is considered gender-neutral, an alternative has not been suggested at this point. The word perinatal is sometimes used, but definitions of maternal health are often considered to encompass the time from conception to 42 days postpartum (WHO, 1992a) whereas perinatal tends to be limited to 22 completed weeks of gestation to 7 days post-birth.			
**Notice for NACI publications with use of additive language** A note on language: NACI recognizes that not all people giving birth or breastfeeding will identify as women or mothers. The writing in this statement uses a gender-additive approach where the term “woman” is used alongside gender-neutral language. This is intended to demonstrate a commitment to redress the historic exclusion of trans and non-binary people, whilst avoiding the risk of marginalizing or erasing the experience of women within the healthcare environment. Finally, NACI acknowledges the dynamic nature of language. It is likely that language deemed to be suitable or affirming in one context may not translate across others, and over the coming years will likely change and evolve with respect to appropriate representations. **Notice for NACI publications with use of additive language and where primary research is reported** A note on language: NACI recognizes that not all people giving birth or breastfeeding will identify as women or mothers. The writing in this statement uses a gender-additive approach where the term “woman” is used alongside gender-neutral language. This is intended to demonstrate a commitment to redress the historic exclusion of trans and non-binary people, whilst avoiding the risk of marginalizing or erasing the experience of women within the healthcare environment. In addition, much of the research available currently refers only to “women” when discussing pregnancy. When citing research, NACI refers to the language used in the study. In these cases, “woman” refers to someone who was assigned female at birth. For the purposes of this statement, the terms “woman,” “women,” should be considered to also apply to those individuals who do not specifically identify as female gender but are the parent gestating the fetus or breastfeeding or chestfeeding the infant. Finally, NACI acknowledges the dynamic nature of language. It is likely that language deemed to be suitable or affirming in one context may not translate across others, and over the coming years will likely change and evolve with respect to appropriate representations. **Notice for CIG chapters with use of additive language and emphasis on language respecting client’s self-identification** Please note: PHAC recognizes that not all people giving birth or breastfeeding will identify as women or mothers. The writing in this chapter uses a gender-additive approach where the term “woman” is used alongside gender-neutral language. This is intended to demonstrate a commitment to redress the historic exclusion of trans and non-binary people, whilst avoiding the risk of marginalizing or erasing the experience of women within the healthcare environment. However, in line with best practice, it is recognized that when discussing or caring for individuals in a one-on-one capacity, language and documentation should reflect the gender identity of the individual. Finally, PHAC acknowledges the dynamic nature of language. It is likely that language deemed to be suitable or affirming in one context may not translate across others, and over the coming years will likely change and evolve with respect to appropriate representations.			
**Tailored Approach:** To convey the importance of inclusivity and representation while not using gender-additive language.			
**WHEN TO USE…** NACI publications that mainly include primary research content or CIG chapters that do not include additive language.			
Try this… (selection of examples)		Instead of this…	Because…
**Notice for publications without use of additive language and where primary research is reported** A note on language: NACI recognizes that not all people giving birth or breastfeeding will identify as women or mothers. Much of the research available currently refers only to “women” when discussing pregnancy. When citing research, NACI refers to the language used in the study. In these cases, “woman” refers to someone who was assigned female at birth. For the purposes of this statement, the terms “woman,” and “women,” should be considered to also apply to those individuals who do not specifically identify as female gender but are the parent gestating the fetus or breastfeeding or chestfeeding the infant. Finally, NACI acknowledges the dynamic nature of language. It is likely that language deemed to be suitable or affirming in one context may not translate across others, and over the coming years will likely change and evolve with respect to appropriate representations. Notice for publications without use of additive language and where emphasis is on the healthcare provider’s use of language that respects a client’s self-identification (e.g., CIG chapters without the use of additive language). Please note: PHAC recognizes that not all people giving birth or breastfeeding will identify as women or mothers. For the purposes of this chapter, the terms “woman”, “women” and “mother” are used but should be considered to also apply to those individuals who do not specifically identify as female gender but are the parent gestating the fetus or breastfeeding or chestfeeding the infant. However, in line with best practice it is recognized that when discussing or caring for individuals in a one-on-one capacity language and documentation should reflect the gender identity of the individual. Finally, PHAC acknowledges the dynamic nature of language. It is likely that language deemed to be suitable or affirming in one context may not translate across others, and over the coming years will likely change and evolve with respect to appropriate representations.		Use of gender-neutral language to replace sexed language.	Depending on the nature and context of the publication it may be most appropriate to include a notice acknowledging the key relevant language issues. For publications where additive language is not yet feasible or in the case of research, not accurately reflective of the language of the study, a clear notice can be used to preface the content with the relevant explanation. In some instances, it may be important to add the recognition that in line with best practices when discussing or caring for individuals in a one-on-one capacity language and documentation should reflect the gender identity of the individual. The aim of this notice is to address inclusivity and, in some cases, to acknowledge an ongoing commitment to finding equitable language solutions.

Abbreviations: CIG, Canadian Immunization Guide; NACI, National Advisory Committee on Immunization; PHAC, Public Health Agency of Canada; Tdap, tetanus, diphtheria, and pertussis; WHO, World Health Organization
